# Effects of Leaf Nutrients, Non-Structural Carbohydrates, and Microanatomical Structure on Biomass of Three Tree Species Under Drought Stress

**DOI:** 10.3390/biology15080629

**Published:** 2026-04-16

**Authors:** Zhaoqun Ma, Xi Zhang, Mengyun Lei, Nan Qin, Wenfang Ma, Lu Han, Haizhen Wang

**Affiliations:** 1 School of Life Science and Technology, Tarim University, Alar 843300, China; 2 School of Agriculture, Tarim University, Alar 843300, China; 3 School of Horticulture and Forestry, Tarim University, Alar 843300, China

**Keywords:** drought stress, carbon–nitrogen metabolism, leaf microanatomical structure, drought-resistance strategy

## Abstract

Drought stress profoundly affects plant growth and survival, but comparisons of integrated adaptive strategies across multiple tree species remain unclear. In this study, seedlings of *Elaeagnus angustifolia* (*E. angustifolia*), *Populus euphratica* (*P. euphratica*) and *Xanthoceras sorbifolium* (*X. sorbifolium*) were subjected to well-watered (CK), mild (T1), moderate (T2), and severe (T3) drought treatments. Leaf microanatomical traits, non-structural carbohydrates (NSCs), stoichiometric elements, biomass allocation, and key stress indicators were measured. The results showed that *P. euphratica* seedlings thickened leaves and vascular tissues and accumulated soluble sugars (SSs) and starch (ST) under T1–T2, but under T3, they prioritized root investment (root biomass +26.0%); their antioxidant enzymes were activated only under mild-to-moderate stress and declined under severe stress. *E. angustifolia* seedlings exhibited moderate leaf structural thickening, sharply increased root biomass (+97.2% under T3) while maintaining stem biomass, continuously elevated activities of superoxide dismutase (SOD) and peroxidase (POD) as well as osmoregulatory substances (soluble protein SP, proline Pro), and showed the lowest malondialdehyde (MDA) content; their leaf carbon (C), nitrogen (N), and phosphorus (P) contents decreased the least, and their stoichiometric ratios remained stable. In contrast, *X. sorbifolium* seedlings progressively reduced leaf thickness and vascular area, depleted NSC reserves, exhibited unstable antioxidant responses, showed a significant decrease in Pro under severe drought, accumulated the highest MDA, and had the lowest N/P ratio, indicating the strongest nitrogen limitation. These results demonstrate that *E. angustifolia* combines structural plasticity, efficient nutrient use, robust osmotic adjustment, and sustained antioxidant capacity, conferring the strongest drought tolerance; P. euphratica* shows moderate tolerance through transient structural and carbon investment but suffers under extreme drought; *X. sorbifolium* has the weakest drought tolerance.

## Simple Summary

Drought severely affects the growth and survival of trees, but it remains unclear how different tree species respond to drought by adjusting their structure, nutrient allocation and internal defense mechanisms. In this study, seedlings of *Elaeagnus angustifolia* (*E. angustifolia*), *Populus euphratica* (*P. euphratica*) and *Xanthoceras sorbifolium* (*X. sorbifolium*) were subjected to various drought gradients; leaf structure, carbon and nitrogen metabolism, biomass allocation, and stress-resistant physiological indices were measured. The results showed that the three species exhibited distinct response patterns to drought: *E. angustifolia* had strong structural plasticity, stable nutrient status, outstanding osmotic adjustment and antioxidant capacity, and thus the strongest drought tolerance; *P. euphratica* showed good adaptability in structure and carbon metabolism under mild to moderate drought but was damaged under severe drought, with moderate drought tolerance; *X. sorbifolium* was weak in structural adjustment, carbon storage and physiological regulation, strongly limited by nitrogen, and had the poorest drought tolerance.

## 1. Introduction

Against the backdrop of global climate change, extreme drought events are becoming more frequent and severe, which not only significantly reduces vegetation productivity but also further alters the composition and geographical distribution of biological communities [1,2]. Under long-term drought stress, plants acclimate to changing environments by regulating a series of complex morphological and functional traits [3,4]. As the primary organ for photosynthesis and transpiration, the leaf is highly plastic and sensitive to changes in the external environment [5]. To maintain internal water balance, plants undergo adaptive changes in leaf morphology and structure, such as increasing the thickness of the adaxial and abaxial epidermis, cuticle, and palisade tissue, thereby reducing water evaporation and enhancing drought tolerance [6].

Non-structural carbohydrates (NSCs) are the main form of carbon storage in plants. They consist of soluble sugars (which help maintain cellular osmotic pressure and defend against stress) and starch (which serves a storage function) [7]. NSCs can act as a carbon buffer when photosynthetic products are insufficient, temporarily supporting plant growth and metabolism. Short-term drought arrests growth and NSC accumulation in seedlings, while prolonged severe drought may lead to net carbohydrate loss. When environmental stress occurs, plants adjust the content and composition of NSC to alter the allocation of energy substances for survival [8]. In addition, nutrients such as nitrogen (N) and phosphorus (P) are essential elements for photosynthesis. They are key components of photosynthetic enzymes and energy metabolism, closely related to photosynthetic efficiency, growth rate, and dry matter production. Their contents and stoichiometric ratios (e.g., N:P) not only affect photosynthetic rate and carbon assimilation capacity but also reflect nutrient limitation status and growth strategies of plants [9,10]. Drought stress induces a series of physiological responses, and plants maintain cell turgor and membrane stability by regulating osmotic adjustment substances (e.g., proline, soluble sugars) and antioxidant enzyme systems, thereby mitigating oxidative damage caused by stress [11]. Therefore, systematic studies on leaf morphological structure, carbon–nitrogen metabolism, physiological indices, and their interrelationships are of great significance for understanding the resource allocation and ecological strategies of afforestation tree species in arid desert areas.

At present, the extensive planting of *Populus euphratica* (*P. euphratica*) in shelterbelts has led to a simple stand structure, while large areas of *P. euphratica* forest have declined and degraded due to a drop in the groundwater table [12]. *Xanthoceras sorbifolium* (*X. sorbifolium*) is both drought-tolerant and of high economic value. Since 2016, Liangzhouhu Town in Manas County has continuously summarized trial planting experience and planted *X. sorbifolium* on barren slopes and wastelands, reaching an area of 467 hm^2^ (over 7000 mu). The introduction and cultivation of *X. sorbifolium* have achieved remarkable results, and its planting has expanded to Manas County, Hutubi County, Fukang City, Jimusaer County, Shihezi, Altay, Aksu, Kashgar Prefecture, Ili and other areas [13]. When selecting drought-tolerant tree species, priority should be given to local provenances or seedlings from provenances whose climatic and site conditions are basically similar to those of the planting site. As a native tree species of Xinjiang, *Elaeagnus angustifolia* (*E. angustifolia*) not only meets this criterion but also possesses excellent biological and ecological characteristics such as drought tolerance, low-temperature tolerance, salt–alkali tolerance, and low nutrient requirements [14]. Therefore, these three tree species were selected as the research subjects, aiming to lay a theoretical foundation for mixed planting of the three species and to mitigate the risk of ecological loss of *P. euphratica*.

Current research on seedlings of these three species has mostly focused on provenance genetics, physiological characteristics, and response mechanisms under stress conditions. However, systematic studies integrating leaf structure, carbon–nitrogen metabolism, and stress physiology to reveal the synergistic relationships among these three aspects and the interspecific differences in adaptive strategies remain insufficient. Based on this, the present study simulated drought stress through a pot-controlled water experiment. We measured the leaf microanatomical structure of the three tree species seedlings, combined with leaf stoichiometric characteristics, NSC dynamics, and stress-related physiological indices, and explored the adaptive strategies and drought tolerance differences in the three species from the perspectives of structure, carbon–nitrogen metabolism, and stress physiology. The aim is to provide a scientific basis for vegetation restoration, afforestation species selection, and structural configuration in arid desert areas.

## 2. Materials and Methods

### 2.1. Study Site Description

The experiment was conducted in 2024 at the Horticultural Experiment Station of Tarim University, located in Alar City, Xinjiang, China, on the northern margin of the Tarim Basin (81°29′ E, 40°54′ N). The region features a warm temperate extreme continental arid desert climate, with an average altitude of 1100 m. Mean annual precipitation ranges from 40.1 to 82.5 mm, and mean annual sunshine duration ranges from 2556.3 to 2991.8 h. The annual mean temperature is 10.7 °C, with an accumulated temperature of ≥10 °C reaching 4113 °C, and a frost-free period of 220 d.

### 2.2. Experimental Design

Healthy two-year-old seedlings of *Populus euphratica*, *Elaeagnus angustifolia*, and *Xanthoceras sorbifolium* were selected from the Horticultural Experiment Station of Tarim University for pot experiments. The bottom of each planting barrel (28 cm in diameter, 30 cm in height) was layered with cobblestones, and water was supplied through a PV pipe installed on the sidewall to avoid surface evaporation. Each barrel was filled with 15 kg of sieved brown calcareous soil, which had a field capacity of 21.90%, a bulk density of 1.47 g/cm^3^, a pH of 7.69, an electrical conductivity of 1511.8 μS/cm, and an organic matter content of 7.59 g/kg. After planting, the seedlings were placed under a rain shelter and routinely watered for recovery. Following the recovery period, a completely randomized block design was employed to impose water stress treatments starting on 15 June 2024. Four drought stress gradients were established for each of the three species: 75–80% *θf* (control, where *θf* represents field capacity), 60–65% *θf* (T1), 45–50% *θf* (T2), and 30–35% *θf* (T3). For each of the three tree species, six replicates (pots) were set up per treatment, with three seedlings per pot. Water was replenished daily by the weighing method to maintain the target water content. During the stress period, the seedlings were exposed to natural sunlight on sunny days and moved into the rain shelter on rainy days. The destructive sampling was conducted on 15 August 2024.

### 2.3. Methods

#### 2.3.1. Determination of Leaf Microanatomical Structure

The microanatomical structure of leaves was analyzed following the method described by Yao et al., with minor modifications [15].

(1) Sampling and sectioning site

For each treatment, the third to fifth fully expanded functional leaves from the top of the seedling (at the mature developmental stage) were collected. Three leaves per plant were pooled to form one replicate, with a total of eight replicates. Tissue blocks of approximately 0.5 cm × 0.5 cm were excised from the middle part of the leaf (avoiding the midrib), perpendicular to the midrib direction, for section preparation.

(2) Fixation and paraffin embedding

Samples were fixed in FAA solution (70% ethanol:formaldehyde:glacial acetic acid = 90:5:5) for more than 24 h and stored at 4 °C. Dehydration in graded ethanol and clearing in xylene were performed using a Leica TP1020 automatic tissue processor (Leica Biosystems Nussloch GmbH, Nussloch, Germany). The samples were then infiltrated with pure paraffin (2 h per step) and embedded in a paraffin:beeswax mixture (10:1) using an embedding station. Both paraffin and beeswax were purchased from Sinopharm Chemical Reagent Co., Ltd., Shanghai, China.

(3) Sectioning and staining

Sections were cut at a thickness of 8–12 μm using a Leica RM2245 semi-automatic rotary microtome (Leica Biosystems Nussloch GmbH, Nussloch, Germany). Staining was performed with 1% safranin (5–10 min) and 0.5% fast green (5–10 s); the dyes were purchased from Shanghai Yuanye Bio-Technology Co., Ltd. (Shanghai, China). After staining, sections were mounted with Canada balsam (Beijing Biolab Technology Co., Ltd., Beijing, China). Section dehydration and drying were carried out using a GZX-9240 MBE forced air drying oven (Shanghai Boxun Industrial Co., Ltd., Shanghai, China).

(4) Observation and measurement

Observations and photomicrographs were taken using an Olympus D2000 optical microscope (Olympus Corporation, Tokyo, Japan) at a magnification of 10 × 10. The OPLENIC_Pro V2.07 software was used to measure leaf lamina thickness (LT), upper and lower epidermal thickness (UET, LET), midrib thickness (MT), upper and lower cuticle thickness (UCT, LCT), palisade parenchyma thickness (Pt), spongy parenchyma thickness (St), midrib vascular bundle area (MVBA), phloem area (MPA), and xylem area (MXA). Based on these measurements, the palisade-to-spongy tissue ratio (P/S), leaf tissue tightness ratio (CTR), and leaf tissue sponginess ratio (SR) were calculated.

P/S = Pt/St(1)

CTR = (Pt/LT) × 100%(2)

SR = (St/LT) × 100%(3)

#### 2.3.2. Determination of Non-Structural Carbohydrates and Stoichiometric Characteristics

After the stress treatment, the potting soil was rinsed with running water. The cleaned seedlings were immediately brought back to the laboratory, where roots, stems, and leaves were separated. Three replicates were set per treatment, with each pot serving as one replicate. Leaf samples were placed into envelopes and subjected to 105 °C for 30 min in an oven (model: DHG-9023A, Shanghai Yiheng Scientific Instrument Co., Ltd., Shanghai, China) to inactivate enzymes, followed by drying at 80 °C to constant weight. The dried samples were ground and passed through a 60-mesh sieve for the determination of soluble sugar, starch, and C, N, P, and K contents. All the above-mentioned required reagents were purchased from Shanghai Yuanye Bio-Technology Co., Ltd.

1. The contents of soluble sugar and starch were determined using a modified phenol-sulfuric acid method [15,16].

(1) Soluble sugar extraction: A total of 40 mg of dried sample was weighed and extracted with 10 mL of 80% ethanol for 24 h. The mixture was centrifuged using a H2-16KR high-speed refrigerated centrifuge (Hunan Kecheng Instrument Equipment Co., Ltd., Hunan, China) at 4000 r·min^−1^ for 10 min, and the supernatant was collected. The precipitate was re-extracted with 5 mL of 80% ethanol and centrifuged for 5 min. The supernatants were combined and diluted to a fixed volume for further analysis.

(2) Starch extraction: The precipitate after the above centrifugation was dried and then mixed with 10 mL of deionized water for gelatinization for 15 min. After cooling, 1 mL of amylase solution was added, and the mixture was incubated until a negative iodine-potassium iodide test was achieved. The enzyme was then inactivated by heating, and the mixture was centrifuged at 2000 r·min^−1^ for 5 min. The supernatant was filtered and diluted to 25 mL.

(3) Determination of soluble sugar and starch concentrations: A total of 1 mL of the test solution was mixed with 1 mL of 28% phenol and 5 mL of concentrated sulfuric acid. After shaking and standing for 15 min, the absorbance was measured at 490 nm using a V-5600PC spectrophotometer (Shanghai Yuanxi Instrument Co., Ltd., Shanghai, China).

2. Determination of leaf stoichiometric characteristics

A 0.2 g aliquot of dried sample was weighed (three replicates), moistened with a small amount of distilled water, and then digested with 5 mL of concentrated H_2_SO_4_ on an SPH108 digestion furnace (Alva Instrument Co., Ltd., Jinan, China) for 30 min. After cooling, 1 mL of 30% H_2_O_2_ was added, and the digestion continued until the solution became colorless and transparent. The digest was diluted to 100 mL, and the supernatant was used for the determination of C, N, P, and K contents. Total nitrogen (N) was determined using a KN520 automatic Kjeldahl nitrogen analyzer (Alva Instrument Co., Ltd., Jinan, China). Total phosphorus (P) content was measured using a 5600PC spectrophotometer (Shanghai Yuanxi Instrument Co., Ltd., Shanghai, China) at a wavelength of 700 nm. Potassium (K) content was determined using an FP6431 flame photometer (Shanghai Yidian Analytical Instrument Co., Ltd., Shanghai, China). Carbon (C) content was determined using the potassium dichromate oxidation-external heating method [17].

#### 2.3.3. Determination of Leaf Physiological Characteristics

The third to fifth fully expanded functional leaves (mature developmental stage) from the apex of seedlings under different water treatments were collected for each of the three species. The leaves were placed in self-sealing bags, transported to the laboratory in an ice box, rinsed thoroughly, and blotted dry with filter paper. They were then stored at −80 °C for subsequent physiological analyses.

Proline content was determined using the ninhydrin method, and absorbance was measured at 520 nm using a 5600PC spectrophotometer (Shanghai Yuanxi Instrument Co., Ltd., Shanghai, China). Malondialdehyde (MDA) content was determined using the thiobarbituric acid method, with absorbance measured at 450, 532, and 600 nm. Superoxide dismutase (SOD) activity was assayed using the NBT reduction method, with absorbance measured at 560 nm. Peroxidase (POD) activity was determined using the guaiacol oxidation method, with absorbance measured at 470 nm, with three to four replicates per treatment [18]. All the above-mentioned required reagents were purchased from Shanghai Yuanye Bio-Technology Co., Ltd.

#### 2.3.4. Biomass Determination

The potting soil was rinsed with running water, taking care to avoid damaging the seedling roots. The seedlings were carefully removed from the pots, immediately brought back to the laboratory, and cleaned thoroughly. Three replicates were set per treatment, with each pot serving as one replicate. Each plant was separated into roots, stems, and leaves. The organs were placed into envelopes and subjected to 105 °C for 30 min in an oven to inactivate enzymes, followed by drying at 80 °C to constant weight. The dry weight of each organ was used to characterize biomass.

### 2.4. Statistical Analysis

Microsoft Excel 2019 was used for data organization, SPSS 26 was used for one-way analysis of variance (ANOVA) and data standardization, and Origin 2024 was used for chart preparation. Principal component analysis was plotted using the Origin 2024 Principal Component Analysis v1.63 plugin.

## 3. Results

### 3.1. Effects of Drought Stress on Leaf Microanatomical Structure of Seedlings

**Figure 1:**
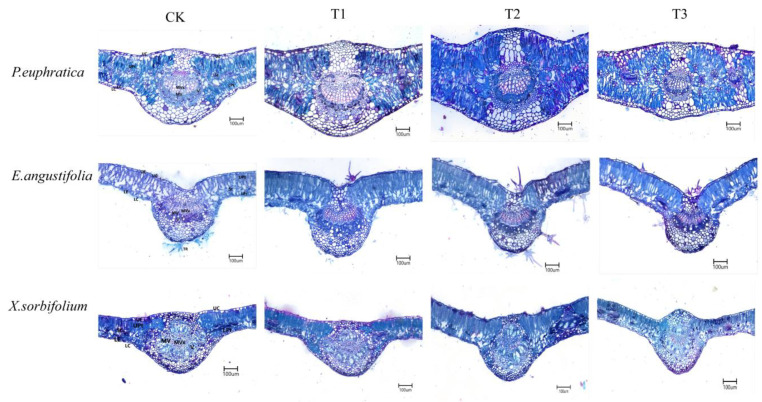
Microanatomical structure of leaves of three seedlings under different drought stress. UC, upper cuticle; LC, lower cuticle; UE, upper epidermis; LE, lower epidermis; UPt, upper palisade parenchyma thickness; LPt, lower palisade parenchyma thickness; St, spongy parenchyma thickness; TR, trichome; MV, main vein (midvein); MVx, midvein xylem; V, vessel. CK represents the well-watered treatment (control), T1 represents mild drought stress, T2 represents moderate drought stress, and T3 represents severe drought stress.

Microscopic images of leaf microanatomical structures (10 × 10 magnification) are shown in Figure 1. The leaf microanatomical structures of the three seedling species exhibited clear hierarchical differentiation, mainly comprising basic tissue layers such as the upper epidermis, palisade tissue, spongy tissue, and lower epidermis. Notably, *E. angustifolia* leaves possessed trichomes; *P. euphratica* leaves exhibited multiple epidermis and contained distinct mucilage cells, with compactly arranged palisade tissue; *X. sorbifolium* leaves were relatively thin but had a larger main vein vascular bundle area. These structural characteristics directly reflect the differences in structural adaptation strategies of the three seedlings to arid environments.

Under mild and moderate drought stresses (T1, T2), the leaf thickness, midrib thickness, palisade tissue thickness, and vascular system (xylem area, vascular bundle area, and phloem area) of *P. euphratica* seedlings increased significantly (Table 1), indicating that *P. euphratica* enhances water transport and photosynthetic capacity by investing in the structure of conductive tissues and palisade tissue. However, under severe drought stress (T3), the above indicators (particularly midrib thickness and vascular system) decreased sharply (*p* < 0.05), suggesting a threshold in its active regulatory capacity, with growth severely inhibited under extreme drought.

As drought stress intensified, the increases in key functional indicators such as LT and Pt of *E. angustifolia* seedlings were relatively small, remaining largely stable (Table 2). Under severe drought stress (T3), these indicators showed only minor decreases (*p* > 0.05), indicating that *E. angustifolia* exhibits high plasticity in leaf structure and can maintain relatively high physiological function under drought stress. For *X. sorbifolium* seedlings, LT and MT generally showed a decreasing trend (Table 3). Under T3 treatment, Pt, MXA, and MVBA decreased significantly *(p* < 0.05), indicating that drought stress significantly inhibited the development of mesophyll and conductive tissues in *X. sorbifolium* seedlings, thereby limiting individual growth and dry matter accumulation. Under drought stress, the leaf microanatomical structures of the three seedling species differed significantly among water treatments and species (*p* < 0.05, Table 1, Table 2 and Table 3). Under T3 treatment, the magnitude of change relative to the control for LT, cuticle thickness (CT), P/S, MXA, and MVBA was highest in *P. euphratica* among the three species with respect to LT (+21.1%), CT (+3.9%), and P/S (+13.4%), while *X. sorbifolium* showed the largest decreases in MXA (−59.3%) and MVBA (−54.9%) (*p* < 0.05). These results indicate that *P. euphratica* adapts to drought stress by developing well-developed mesophyll tissue and vascular systems to maintain photosynthetic gas exchange and water transport.

### 3.2. Effects of Drought Stress on Non-Structural Carbohydrates in Seedling Leaves

**Figure 2:**
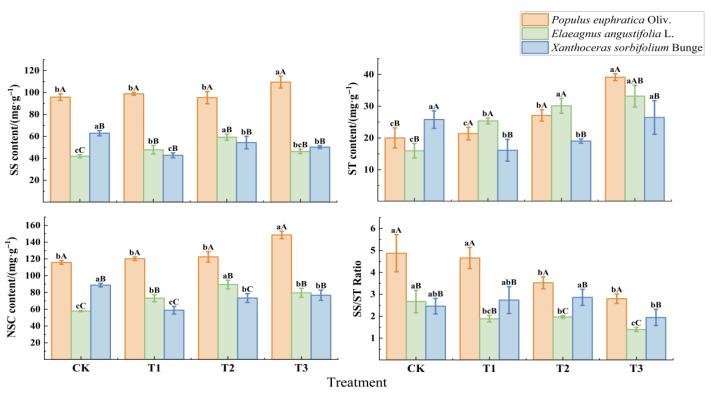
Response of the leaf NSC to drought stress in different species. All experiments were performed in triplicate. Different lowercase letters indicate significant differences (*p* < 0.05) among different stress treatments for the same tree species at the same stress time; different uppercase letters indicate significant differences (*p* < 0.05) among the same stress treatment for different tree species at the same stress time. CK represents the well-watered treatment (control), T1 represents mild drought stress, T2 represents moderate drought stress, and T3 represents severe drought stress.

Under drought stress, the contents of non-structural carbohydrates (NSCs), soluble sugars (SSs), and starch (ST), as well as the SS/ST ratio in leaves of the three seedling species, exhibited distinct trends with increasing drought intensity, with significant interspecific differences (*p* < 0.05, Figure 2). Leaf SS and NSC contents and the SS/ST ratio in *P. euphratica* were significantly higher than those in *E. angustifolia* and *X. sorbifolium* (*p* < 0.05).

With increasing drought stress, NSC and SS contents in *P. euphratica* seedlings increased continuously, rising by 28.3% and 14.2%, respectively, under T3 compared with CK (*p* < 0.05); ST content also increased significantly (*p* < 0.05). In *E. angustifolia* seedlings, NSC and SS contents initially increased and then decreased, both peaking under T2 (*p* < 0.05), and remained at relatively high levels under T3; ST content accumulated progressively with increasing drought stress (*p* < 0.05). In *X. sorbifolium* seedlings, NSC, SS, and ST contents were consistently lower than those in CK throughout the drought stress period; ST content decreased significantly already under T1 (*p* < 0.05), while NSC and SS contents reached their lowest values under T3 (*p* < 0.05).

### 3.3. Effects of Drought Stress on Leaf Stoichiometric Characteristics

**Figure 3:**
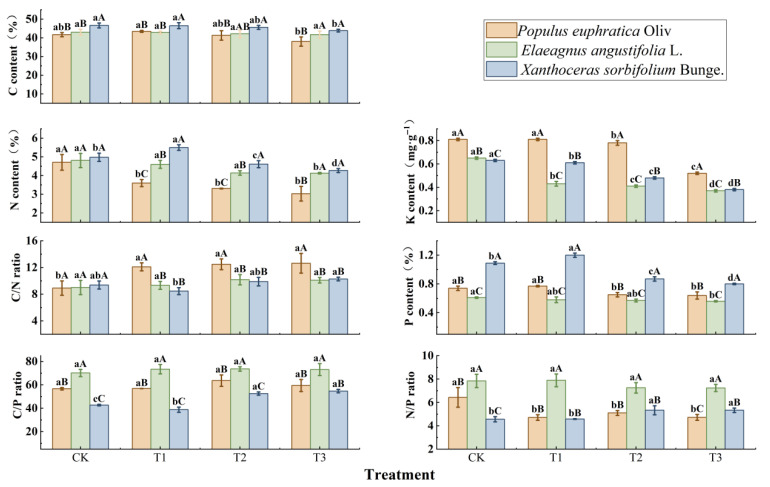
Comparison of leaf stoichiometric characteristics among three tree species seedlings under different drought treatments. All experiments were performed in triplicate. Different lowercase letters indicate significant differences (*p* < 0.05) among different stress treatments for the same tree species at the same stress time; different uppercase letters indicate significant differences (*p* < 0.05) among the same stress treatment for different tree species at the same stress time. CK represents the well-watered treatment (control), T1 represents mild drought stress, T2 represents moderate drought stress, and T3 represents severe drought stress.

Under different water treatments, the leaf stoichiometric characteristics of the three tree species seedlings showed significant differences (*p* < 0.05, Figure 3). The leaf carbon (C), nitrogen (N), and phosphorus (P) contents of *X. sorbifolium* were significantly higher than those of the other two species, while its C/P ratio showed the opposite trend. With increasing drought stress, the C, N, P, and potassium (K) contents and stoichiometric ratios of the three species exhibited different trends. The C/P and N/P ratios of *X. sorbifolium* increased significantly with drought intensity, with C/P rising from 42.72 ± 0.64 under CK to 54.73 ± 1.46 under T3 (*p* < 0.05), and N/P increasing from 4.56 ± 0.22 to 5.33 ± 0.19 (*p* < 0.05), and the N/P ratio of *X. sorbifolium* was the lowest among the three species, indicating that the growth of *X. sorbifolium* was most strongly affected by nitrogen limitation. In *E. angustifolia*, the decreases in C, N, and P contents were the smallest among the three species, and no significant differences were observed in C/P, C/N, or N/P ratios under different drought treatments, while its C/P and N/P ratios were significantly higher than those of the other two species (*p* < 0.05). The leaf K content of *P. euphratica* was significantly higher than that of the other two species. Its leaf N content was 4.71 ± 0.42 g·kg^−1^ under CK and decreased to 3.03 ± 0.39 g·kg^−1^ under T3, significantly lower than that of the other two species (*p* < 0.05). The C/N ratio increased with drought intensity, while the N/P ratio decreased, suggesting that the growth of *P. euphratica* was also limited by nitrogen under drought stress. Under different water treatments, the leaf N/P ratios of *P. euphratica*, *E. angustifolia*, and *X. sorbifolium* were all well below 14, the global average threshold for plants.

### 3.4. Effects of Drought Stress on Leaf Biomass of Different Tree Species Seedlings

**Figure 4:**
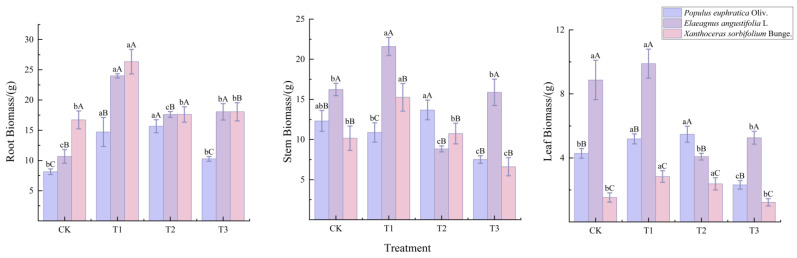
Changes in leaf biomass of three tree species seedlings under different drought treatments. Notes: All experiments were performed in triplicate. Different lowercase letters indicate significant differences (*p* < 0.05) among different stress treatments for the same tree species at the same stress time; different uppercase letters indicate significant differences (*p* < 0.05) among the same stress treatment for different tree species at the same stress time. CK represented the well-watered treatment (control), T1 represented mild drought stress, T2 represented moderate drought stress, and T3 represented severe drought stress.

As shown in Figure 4, leaf biomass differed significantly among the three seedling species under drought stress (*p* < 0.05). Compared with the control (CK), leaf biomass of all seedlings decreased significantly under T3 (*p* < 0.05), while root biomass increased significantly. Stem biomass decreased significantly in *P. euphratica* and *X. sorbifolium*, but showed no significant change in *E. angustifolia.* Under T1, leaf biomass of *E. angustifolia* and *X. sorbifolium* was significantly higher than that under other treatments; under T2, leaf biomass of *P. euphratica* was significantly higher than that under other treatments.

Under severe drought stress (T3), the magnitudes of change in root, stem, and leaf biomass relative to the control varied significantly among the three species, reflecting distinct biomass allocation strategies. *P. euphratica* showed a 26.0% increase in root biomass, accompanied by decreases of 39.0% and 45.8% in stem and leaf biomass, respectively, representing a typical drought-avoidance strategy that prioritizes resource allocation to roots while substantially reducing aboveground parts to minimize water loss. *E. angustifolia* exhibited a substantial increase in root biomass (97.2%), relatively stable stem biomass (only 2.2% decrease), and a 40.7% decrease in leaf biomass, demonstrating a root–leaf co-maintenance drought-tolerance strategy that enhances water uptake while preserving photosynthetic capacity. *X. sorbifolium* showed relatively smaller changes across organs, with root biomass increasing by 8.0%, and stem and leaf biomass decreasing by 34.9% and 19.6%, respectively.

### 3.5. Effects of Drought Stress on Leaf Physiological Characteristics of Seedlings of Different Tree Species

**Figure 5:**
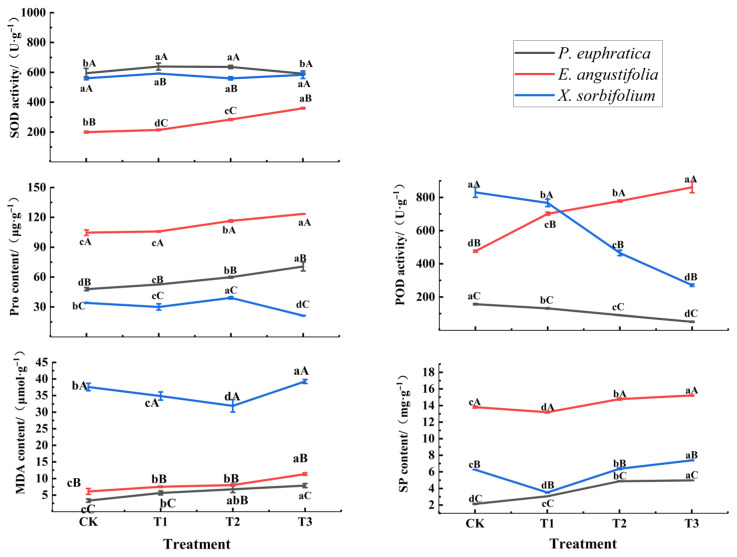
Comparative leaf physiological traits in seedlings of three tree species under different drought treatments. Notes: All experiments were performed in triplicate. Different lowercase letters indicate significant differences (*p* < 0.05) among different stress treatments for the same tree species at the same stress time; different uppercase letters indicate significant differences (*p* < 0.05) among the same stress treatment for different tree species at the same stress time. CK represents the well-watered treatment (control), T1 represents mild drought stress, T2 represents moderate drought stress, and T3 represents severe drought stress.

As shown in Figure 5, with increasing drought stress, the seedlings of the three tree species exhibited different response strategies in terms of antioxidant enzyme activities and osmoregulatory substances. Regarding antioxidant enzymes, SOD activity in *P. euphratica* was significantly higher under T1 and T2 treatments compared with CK, but decreased to the CK level under T3 treatment, while POD activity continuously decreased with increasing stress. In contrast, SOD and POD activities in *E. angustifolia* continuously increased with stress intensity, increasing by 79.9% and 81.1%, respectively, compared with CK under the T3 treatment. For *X. sorbifolium*, SOD activity remained relatively stable across treatments, while POD activity initially decreased, then increased, and finally decreased again. Regarding osmoregulatory substances, SP and Pro contents in *P. euphratica* and *E. angustifolia* continuously increased with stress intensity, with *E. angustifolia* exhibiting significantly higher SP and Pro contents than the other two species across all treatments (*p* < 0.05). In *X. sorbifolium*, SP content generally increased, but Pro content significantly decreased by 37.6% under the T3 treatment compared with CK. Regarding membrane lipid peroxidation, MDA content in the three species generally increased with stress intensity, with *X. sorbifolium* showing significantly higher MDA content than *P. euphratica* and *E. angustifolia* across all treatments (*p* < 0.05), indicating the most severe degree of membrane lipid peroxidation. In summary, *E. angustifolia* exhibited strong responsiveness in both antioxidant enzyme activity and osmoregulatory capacity, whereas *X. sorbifolium* showed higher levels of membrane lipid peroxidation and a relatively weaker antioxidant system response.

### 3.6. Interrelationships Between Leaf Structure and Physiological Metabolism in Seedlings of Three Tree Species Under Drought Stress

**Figure 6:**
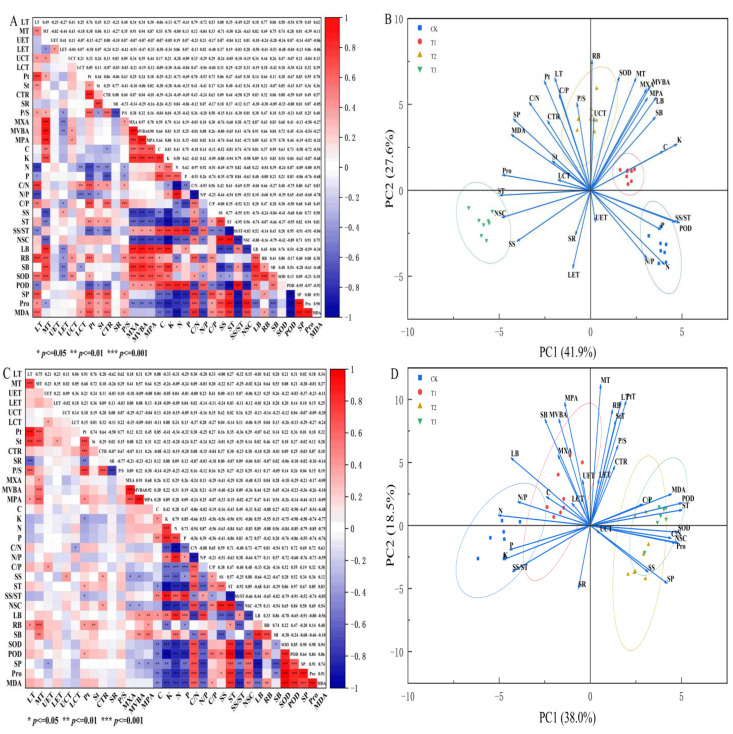

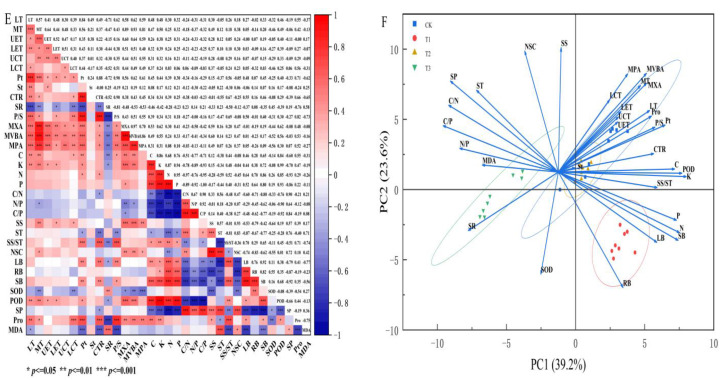
Correlation and principal component analysis results of various indicators in three seedling species under. Note: LT for leaf lamina thickness; MT for midrib thickness; UET for upper epidermal thickness; LET for lower epidermal thickness; UCT for upper cuticular thickness; LCT for lower cuticular thickness; Pt for palisade parenchyma thickness, St for spongy parenchyma thickness; CTR for leaf tissue compactness; SR for leaf tissue sponginess; P/S for palisade-to-spongy tissue ratio; MXA for xylem area; MVBA for midrib vascular bundle area; MPA for phloem area; C for carbon content; K for potassium content; N for nitrogen content; P for phosphorus content; C/P for carbon-to-phosphorus ratio; C/N for carbon-to-nitrogen ratio; N/P for nitrogen-to-phosphorus ratio; SS for soluble sugar content; ST for soluble starch content; SS/ST for soluble sugar-to-starch ratio; NSC for non-structural carbohydrate content; BM for biomass. SP for soluble protein, Pro for proline, MDA for malondialdehyde, SOD for superoxide dismutase, POD for peroxidase, RB for root biomass, SB for stem biomass, LB for leaf biomass. CK represents the well-watered treatment (control), T1 represents mild drought stress, T2 represents moderate drought stress, and T3 represents severe drought stress. (**A**,**B**) represent *P. euphratica* seedlings, (**C**,**D**) represent *E. angustifolia* seedlings, and (**E**,**F**) represent *X. sorbifolium* seedlings.

Under drought stress, most leaf microanatomical structure indicators—such as LT, MT, UCT, and PtT—exhibited significant positive correlations among the three seedling species (Figure 6A,C,E). This suggests a coordinated integration of leaf morphological construction under drought conditions, with structural synergy representing one of the adaptive strategies to drought. Certain leaf structural parameters (LT, MT, PtT, P/S, StT, CTR, UCT, and MXA) were significantly correlated with NSC, SS, ST, SS/ST, C, C/N, C/P, and N/P, indicating a synergistic adaptation strategy involving water transport, defensive structures, mesophyll tissue construction, and carbon and nitrogen metabolism. However, the adaptive responses to drought stress differed among the three species (Figure 6B,D,F).

As shown in Figure 6A, the biomass and SOD activity of *P. euphratica* seedlings showed highly significant positive correlations with vascular bundle structure and midrib thickness (r = 0.63~0.91, *p* < 0.001), while soluble sugar content exhibited a highly significant negative correlation with vascular bundle structure (r = −0.74~−0.80, *p* < 0.001). Starch and non-structural carbohydrates were also highly significantly negatively correlated with both vascular bundle structure and stoichiometric elements (r = −0.63~−0.98, *p* < 0.001). Meanwhile, soluble protein, proline, and malondialdehyde contents showed highly significant negative correlations with stoichiometric elements, N/P ratio, and the SS/ST ratio (r = −0.54~−0.91, *p* < 0.001), but highly significant positive correlations with C/N ratio, C/P ratio, soluble sugar, and starch contents (r = 0.40~0.94, *p* < 0.001). These results indicate that *P. euphratica* seedlings synergistically promote growth and antioxidant defense by strengthening vascular structures, yet must balance carbon reserves (soluble sugar and starch) against structural investment. Furthermore, the balance of carbon and nitrogen metabolism significantly affects osmotic regulation and membrane lipid peroxidation: when carbon is abundant, osmotic substances accumulate, accompanied by increased membrane damage, whereas adequate nutrients help alleviate stress responses.

As shown in Figure 6C, The physiological indices of *E. angustifolia* seedlings showed highly significant negative correlations with stoichiometric characteristics (r = −0.47~−0.97, *p* < 0.001), N/P ratio (r = −0.59~0.76, *p* < 0.001), SS/ST ratio (r = −0.51~−0.91, *p* < 0.001), and LB (r = −0.55~0.90, *p* < 0.001), and highly significant positive correlations with C/N ratio, ST, and NSC (r = 0.53~0.97, *p* < 0.001), but showed weak correlations with microanatomical structural indices. Regarding biomass, total biomass was significantly positively correlated with MVBA and MPA, root and stem biomasses were positively correlated with MT, and aboveground biomass was highly significantly positively correlated with N content and N/P ratio (r = 0.56~0.85, *p* < 0.001). These results indicate that the physiological indices (soluble protein, proline, MDA, etc.) of *E. angustifolia* seedlings are tightly coupled with carbon–nitrogen metabolic balance: when carbon is relatively abundant (high C/N, high starch, high NSC), osmotic substances accumulate accompanied by increased membrane lipid peroxidation, whereas adequate nutrients (N, P) and a high N/P ratio significantly suppress physiological stress indicators. Biomass accumulation mainly depends on the development of vascular tissues (MVBA, MPA) and MT, and aboveground growth is promoted by nitrogen, reflecting a trade-off strategy between carbon reserve and growth investment in *E. angustifolia* seedlings.

As shown in Figure 6E, POD activity showed highly significant positive correlations with stoichiometric elements (r = 0.84~0.99, *p* < 0.001), and highly significant negative correlations with stoichiometric ratios (r = −0.76~−0.92, *p* < 0.001). Biomass was highly significantly positively correlated with N and P contents. Pro was highly significantly positively correlated with vascular bundle structure (MXA, MVBA), P/S, and C and K contents. In addition, POD activity was also highly significantly positively correlated with vascular bundle structure (MXA, MVBA), LT, and MT. This indicates that POD antioxidant enzyme activity is highly synergistic with nutrient elements (especially C and K) and closely coupled with leaf structural development, while proline accumulation also depends on vascular development and carbon–potassium supply, reflecting a coordinated strategy between antioxidant defense and structural investment in *X, sorbifolium* seedlings. Biomass is promoted by both nitrogen and phosphorus, further confirming the direct driving effect of nutrients on growth.

Figure 6B shows that the first two principal components (PC1 and PC2) jointly explained 69.5% of the total variation in *P. euphratica* traits. PC1 was primarily loaded by nutrient contents (C, K, N, P), antioxidant enzyme (POD), and vascular structures, while PC2 was mainly loaded by leaf tissue thickness, root biomass (RB), and SOD. With increasing drought stress, the distribution of seedling traits in the PC1-PC2 space shifted progressively. The control (CK) was located in the high PC1, low PC2 region, representing a resource-acquisition and growth-priority strategy. Mild drought (T1) shifted positively along PC2, with growth inhibition and enhanced structural adaptation. Moderate drought (T2) moved further positively along PC2, adapting by strengthening leaf defensive structures and promoting root growth. Severe drought (T3) shifted rapidly negatively along PC1, adopting a survival strategy based on NSC accumulation, osmotic regulation (Pro, SP), and increased MDA. Thus, as drought intensifies, the resource allocation of *P. euphratica* shifts from a “resource acquisition, growth priority” strategy to a “structural defense, root expansion” strategy, and finally to a “carbon reserve and osmotic regulation” strategy.

PC1 and PC2 cumulatively explained 56.5% of the total variance in leaf traits of *E. angustifolia* (Figure 6D). On the positive axis of PC1, SOD, POD, Pro, NSC, MDA and C/N exhibited high loadings, while K, N and P showed high loadings on the negative axis. On the PC2 axis, LT, MT, PtT, MPA and MVBA had high loadings. The CK treatment displayed a high-mineral-nutrient acquisition strategy. As drought intensified, the T1 treatment shifted towards a less negative PC1 and a positive PC2, increasing investment in membrane protection and vascular tissue structure, indicating a strategy mainly based on structural optimization. The T2 treatment shifted positively along PC1, forming a strategy characterized by high antioxidant enzyme activities, low mineral element contents, and a high C/N reserve. The T3 treatment further strengthened the positive PC1 direction, while the overall PC2 scores shifted positively, indicating that, on the basis of maintaining high antioxidant and osmotic adjustment capacities, it further aggravated membrane lipid peroxidation (MDA, LT) and increased vascular tissue investment (MPA, MVBA), thereby forming a comprehensive stress tolerance strategy.

As shown in Figure 6F, PC1 and PC2 cumulatively explained 62.8% of the total variance in traits of *X. sorbifolium*. On the positive axis of PC1, leaf thickness (LT, MT, PtT, etc.), mineral elements, POD and Pro exhibited high loadings, while stoichiometric ratios and SP showed high loadings on the negative axis. On the positive axis of PC2, vascular tissues (MVBA, MPA) and non-structural carbohydrates had high loadings, whereas SOD and RB showed high loadings on the negative axis. The CK treatment displayed a high-structure-investment and high-non-structural-carbon-reserve strategy. As drought intensified, the T1 treatment remained positive on PC1 but shifted significantly negative on PC2, characterized by maintained leaf thickness and mineral elements but reduced non-structural carbohydrates and increased SOD activity to adapt to water deficit. The T2 treatment showed a PC1 value close to zero and a positive shift on PC2, forming a strategy of moderate structural investment and carbon-reserve recovery. The T3 treatment turned negative on PC1, with overall PC2 scores shifting negatively, indicating that, on the basis of low mineral elements and a high C/N ratio, it further reduced non-structural carbohydrates and increased SOD activity, thereby forming a stress-tolerance strategy dominated by restricted carbon accumulation and antioxidant defense.

## 4. Discussion

### 4.1. Responses of Leaf Microanatomical Structure of Seedlings of Different Tree Species to Drought Stress

Leaf microanatomical structure characteristics provide a more intuitive understanding of how tree species respond to environmental changes and are also important indicators for assessing plant adaptability to the environment [19]. In this study, the leaf structures of seedlings from three tree species showed significantly differentiated characteristics. *P. euphratica* seedlings possessed multiple epidermis and visible mucilage cells, which can store water and reduce transpirational water loss under drought conditions [20]. *E. angustifolia* seedlings had densely distributed trichomes on the leaf surface; these trichomes increase the thickness of the leaf boundary layer and reflect part of the light radiation, thereby reducing leaf surface temperature and transpiration rate [21]. Although the leaves of *X. sorbifolium* were relatively thin, their main vein structure was well developed, with vascular bundles occupying three-quarters of the main vein area, facilitating water transport [22]. The leaf and palisade tissue thickness of *P. euphratica* and *E. angustifolia* seedlings increased with intensifying drought stress. In contrast, the leaf and palisade tissue thickness of *X. sorbifolium* seedlings continuously decreased under increasing drought stress. These results are consistent with the findings of Shi F et al. [18] and Z Kang et al. [23], indicating that under drought stress, *P. euphratica* and *E. angustifolia* seedlings enhance their leaf water retention and protective capacity by thickening the epidermis and mesophyll tissue, whereas *X. sorbifolium* seedlings exhibit thinner leaves and reduced photosynthetic and water retention capacities, leading to growth inhibition and the lowest biomass (Figure 4). Under drought stress, changes in plant leaf microanatomical structure directly affect photosynthetic capacity and water transport efficiency. As the primary site of photosynthesis, an increase in palisade tissue thickness generally enhances light capture and utilization efficiency. Meanwhile, the spongy tissue is responsible for gas exchange and water conduction; changes in its compactness and thickness are related to water storage and transport pathways within the leaf [24,25]. In this study, under severe drought stress, the palisade tissue thickness of *P. euphratica* and *X. sorbifolium* seedlings decreased significantly, potentially inhibiting photosynthetic capacity. In contrast, the palisade tissue thickness of *E. angustifolia* seedlings under severe stress increased compared to that under moderate stress, indicating a tendency to maintain carbon assimilation capacity by enhancing photosynthetic tissue [26,27]. At the same time, the spongy tissue thickness of the three species showed differential responses under different stress levels, with the spongy tissue storing substantial amounts of water. The spongy tissue thickness of *P. euphratica* seedlings first increased and then decreased, peaking at T2 (moderate stress) before declining, suggesting that under moderate drought, it may enhance water storage capacity. The spongy tissue thickness of *X. sorbifolium* showed an overall decreasing trend, indicating a gradual decline in water storage capacity of the leaf spongy tissue under stress. The spongy tissue thickness of *E. angustifolia* showed an overall increasing trend with intensifying stress, indicating that it copes with drought by continuously enhancing leaf water storage capacity [18]. The ability of *E. angustifolia* to maintain or even increase palisade tissue thickness under severe stress may be related to its strong osmotic adjustment capacity (high accumulation of proline and soluble sugars) (Figure 5). Osmotic adjustment helps maintain cell turgor [28], thereby ensuring normal cell division and expansion [29]. The synergistic or divergent changes between palisade and spongy tissues can improve water transport and reduce water loss in each tree species, thereby enhancing photosynthetic productivity [23,30]. As the core structure for water and nutrient transport in leaves, the development of vascular bundles directly reflects plant adaptation strategies to drought stress. Studies have shown that an increase in vascular bundle area or vessel diameter can improve water transport efficiency, but overly large vessels are prone to embolism under drought conditions, increasing the risk of hydraulic failure [31]. In this study, with increasing drought stress, the vascular bundle area of *P. euphratica* and *X. sorbifolium* decreased sharply, indicating that they may adopt a conservative hydraulic strategy by reducing the scale of conductive tissue to lower the risk of water loss. In contrast, the vascular bundle area of *E. angustifolia* remained relatively high under severe stress, reflecting its attempt to maintain water transport efficiency to adapt to drought environments. The differences in vascular bundle structure among the three tree species reveal their different trade-offs between efficiency and safety in water and nutrient transport.

### 4.2. Responses of Leaf Carbon and Nitrogen Metabolism of Different Tree Species Seedlings to Drought Stress

Non-structural carbohydrates (NSCs) serve as important buffering substances for plants responding to environmental changes, playing a significant role in regulating internal carbon balance and resisting stressful environments. Currently, the impact of drought stress on plant NSC content remains inconclusive due to variations in tree species, site conditions, and climate [32,33], Under drought stress, plants can increase soluble sugar (SS) content through two pathways: one is hydrolysis and conversion from starch (ST), and the other is direct synthesis through photosynthesis [34,35]. These two pathways also represent different plant adaptation strategies. In this study, prolonged drought stress significantly increased leaf SS, ST, and NSC contents in *P. euphratica* seedlings while decreasing the SS/ST ratio, indicating that SS in *P. euphratica* leaves was mainly directly synthesized through photosynthesis, while ST accumulation served as a carbon and energy reserve for long-term drought, maintaining internal carbon balance. This aligns with Chen et al. [36], who reported that *Haloxylon ammodendron* maintained photosynthetic carbon fixation under drought conditions, resulting in significantly increased ST and NSC contents. Under drought stress, *E. angustifolia* seedlings showed initial increases followed by decreases in SS and NSC contents, significantly increased ST content, significantly decreased SS/ST ratio, and SS content remained higher than the control group (CK) even under severe drought stress. This suggests two aspects: first, under severe drought stress, after reduced photosynthetic carbon assimilation, *E. angustifolia* preferentially stores carbon in the form of ST to maintain internal carbon balance and energy supply; second, leaves actively increase osmotic adjustment substances such as proline to enhance cellular osmotic potential and promote water absorption [37], reducing dependence on SS. Compared with *P. euphratica* and *E. angustifolia*, *X. sorbifolium* seedlings showed significantly reduced SS, NSC, and ST contents under drought stress, indicating that drought conditions significantly inhibit photosynthetic carbon assimilation. Reduced photosynthetic products combined with increased respiratory consumption [38,39] make NSC accumulation difficult, and intensified drought may lead to carbon starvation and mortality. This also reflects the poor drought resistance of *X. sorbifolium*.

Carbon (C), nitrogen (N), and phosphorus (P) are essential elements constituting plant frameworks and various proteins and nucleic acids, participating in various physiological metabolic processes. Changes in their stoichiometric characteristics not only reflect plant resource allocation strategies and growth status but also indicate plant responses to environmental changes and nutrient limitation. Research found that drought stress had no significant effect on leaf carbon content of the three tree species, mainly because C provides substances such as carbohydrates for plant structure, growth, and reproduction, maintaining relative stability in plants [40]. With increasing drought stress, N, P, and K contents in the three seedlings showed decreasing trends, attributed to reduced soil N, P, and K availability under drought conditions, leading to decreased absorbable nutrients from roots. Reduced soil water content also caused decreased water potential and root activity, resulting in reduced nutrient absorption from the soil and increased resistance to upward nutrient transport [41,42], consequently decreasing leaf N, P, and K^+^ contents. Additionally, *P. euphratica* seedlings showed significantly higher K^+^ content than the other two species, indicating that *P. euphratica* maintains cellular osmotic potential and regulates stomatal opening and closing by increasing intracellular K^+^ content under drought stress, enhancing leaf photosynthetic efficiency and biochemical metabolic activity, thereby producing more organic matter [43], and storing more NSC and its components. Meanwhile, high intracellular K^+^ and SS contents favor maintaining lower leaf water potential, promoting leaf expansion in *P. euphratica* seedlings, manifested as significantly higher structural indicators (LT, CT, P/S, VX, and VBA) compared to the other two species.

C/N and C/P ratios are important indicators reflecting plant N and P nutrient use efficiency, with higher ratios indicating higher efficiency of N and P absorption and assimilation [40]. Our results showed that *P. euphratica* had the highest C/N ratio, while *E. angustifolia* had the highest C/P ratio, indicating relatively high N and P absorption and utilization efficiency in these two species. Meanwhile, *X. sorbifolium* seedlings had significantly higher P content than other species, suggesting higher P demand. Under drought stress, plants adopting efficient nutrient utilization strategies can effectively enhance their drought resistance and survival competitiveness. Plant N/P ratio is commonly considered an indicator of community vegetation composition, function, and nutrient limitation, consistent with research showing that N/P < 14 typically indicates N limitation, while N/P > 16 indicates P limitation [44]. In this study, *E. angustifolia* had the highest and most stable N/P ratio, indicating the weakest N limitation; *X. sorbifolium* had the lowest N/P ratio, indicating the strongest N limitation. Overall, the three tested species had N/P < 14, indicating predominant N limitation under drought stress, with drought further exacerbating N and P nutrient limitation.

### 4.3. Response of Leaf Physiological Characteristics of Different Tree Species Seedlings to Drought Stress

When plants suffer from drought stress, the balance of reactive oxygen species (ROS) metabolism in their tissues is disrupted. Antioxidant enzymes such as SOD and POD can scavenge ROS and maintain normal cellular activities [45]. The SOD and POD activities of *E. angustifolia* seedlings showed a continuous increasing trend with intensifying stress, reaching their maximum under the T3 treatment, indicating that their activities are continuously activated, which helps *E. angustifolia* seedlings scavenge ROS, inhibit membrane lipid peroxidation, and reduce damage to the membrane system. The SOD activity of *P. euphratica* seedlings increased significantly under mild and moderate stress (T1, T2) but decreased to the control level under severe stress (T3), which is consistent with the findings of Qi Y et al. [46], indicating that under severe drought stress, the plant’s defense mechanism is disrupted and exceeds the scavenging capacity of protective enzymes. Meanwhile, the POD activity of *P. euphratica* seedlings continuously decreased with intensifying stress, suggesting that *P. euphratica* can activate SOD to cope with oxidative stress under mild to moderate stress, but under long-term severe stress, its antioxidant enzyme system may be inhibited due to excessive stress [18], making it difficult to maintain SOD and POD activities. The SOD activity of *X. sorbifolium* seedlings remained generally stable across treatments, while POD activity showed fluctuating changes, indicating that the response of its antioxidant enzyme system to water stress is relatively complex. It may rely to some extent on SOD to maintain basal antioxidant capacity, but the POD response is not sufficiently stable.

SP and Pro are important osmotic regulatory substances in plants. Under drought stress, they accumulate to lower the cellular osmotic potential, maintain cell turgor, and enhance the cell’s ability to absorb and retain water, thereby ensuring normal plant growth [18]. In this study, the SP and Pro contents of *E. angustifolia* and *P. euphratica* showed a continuous increasing trend with intensifying stress, consistent with the findings of Hu X et al. [47]. This indicates that both species can adapt to water stress by accumulating osmotic regulatory substances. For *X. sorbifolium*, SP content generally increased with intensifying stress, but Pro content decreased significantly under T3 compared to the control, indicating that under severe stress, proline synthesis may be inhibited or catabolism enhanced, leading to reduced osmotic adjustment capacity [48]. This is consistent with the high MDA content and severe membrane lipid peroxidation observed in *X. sorbifolium*, further demonstrating that its tolerance to severe water stress is relatively weak. The SP and Pro contents of *E. angustifolia* were significantly higher than those of *P. euphratica* and *X. sorbifolium* under all treatments, indicating that it has the strongest osmotic adjustment capacity. This may be an important physiological basis for *E. angustifolia* to maintain relatively better growth performance under drought stress.

MDA is the final product of membrane lipid peroxidation, and its content reflects the degree of cell membrane damage [49]. In this study, the MDA content of seedlings of the three tree species generally increased with intensifying stress, indicating that water stress caused a certain degree of oxidative damage to the cell membranes, which is consistent with the findings of ZHU Y et al. [50]. However, there were significant differences in MDA accumulation among the different tree species: *X. sorbifolium* had significantly higher MDA content under all treatments than *P. euphratica* and *E. angustifolia*, indicating that it suffered the most severe membrane lipid peroxidation and the most obvious cell membrane damage. *E. angustifolia* had relatively low MDA content under all treatments, and the increase under T3 was relatively small, indicating that its cell membrane stability is strong and its antioxidant protection mechanism is relatively effective.

## 5. Conclusions

Drought stress significantly affected the morphological structure and physiological metabolic processes of the seedlings of the three tree species, leading to different adaptive strategies in response to drought stress. *P. euphratica* seedlings actively thickened leaves and vascular tissues and continuously accumulated soluble sugars and starch under mild to moderate drought stress; under severe stress, they prioritized root supply (root biomass +26.0%, with substantial reductions in stem and leaf biomass). Their antioxidant capacity was activated under mild to moderate stress but impaired under severe stress. They maintained a relatively high K^+^ content, while their nitrogen content decreased the most under severe stress, and the C/N ratio increased, indicating that their growth was also limited by nitrogen. Their comprehensive drought tolerance was moderate. *E. angustifolia* seedlings showed moderate thickening of leaf structures; non-structural carbohydrates (NSC) were first accumulated and then mobilized. Under severe stress, root biomass increased sharply by 97.2%, while stem biomass remained stable (only −2.2%). Their SOD and POD activities and osmoregulatory substances (SP, Pro) continuously increased, and the degree of membrane lipid peroxidation was the lowest. Their C, N, and P contents decreased the least, with no significant differences in stoichiometric ratios under different drought treatments, and their C/P and N/P ratios were significantly higher than those of the other two species, indicating the most stable nutrient regulation. Their comprehensive drought tolerance was the strongest. *X. sorbifolium* seedlings had weak active regulation ability; leaf thickness and vascular tissue area continuously decreased, NSC was consistently lower than the control, and carbon reserves were depleted. Their biomass allocation changes were small (root only +8.0%). Their antioxidant system response was unstable, proline decreased significantly under severe stress, and MDA accumulation was the highest. They were most strongly limited by nitrogen (with the lowest N/P ratio among the three species), and their drought tolerance was the weakest. In summary, *X. sorbifolium* has relatively weak drought tolerance and is not recommended for afforestation in extremely arid regions. However, it still possesses high economic value and planting potential in semi-arid areas, barren slopes and wastelands, as well as sites with relatively favorable water conditions. It is recommended to adopt site-specific allocation strategies: prioritize mixed forests of *P. euphratica* and *E. angustifolia* in extremely arid regions, while *X. sorbifolium* can be introduced in areas with better water conditions to balance ecological restoration and economic benefits.

## Figures and Tables

**Table 1:** Effects of drought stress on seedling microanatomical structure of *P. euphratica*.

Index	*P. euphratica*			
	CK	T1	T2	T3
Leaf lamina thickness (μm)	315.77 ± 7.20 cA	389.50 ± 5.26 bA	415.05 ± 8.97 aA	382.28 ± 16.28 bA
Midvein thickness (μm)	589.32 ± 9.59 cA	650.07 ± 6.80 bA	713.49 ± 9.61 aA	512.90 ± 32.37 dA
Upper epidermal thickness (μm)	35.01 ± 1.80 aA	34.09 ± 1.36 aA	32.71 ± 2.07 aA	34.48 ± 2.72 aA
Lower epidermal thickness (μm)	29.22 ± 1.33 aA	28.60 ± 1.31 abA	26.09 ± 1.16 bA	30.35 ± 1.15 aA
Upper stratum corneum thickness (μm)	3.11 ± 0.33 bA	3.71 ± 0.33 abA	4.02 ± 0.39 aA	3.23 ± 0.26 bA
Lower stratum corneum thickness (μm)	2.51 ± 0.30 cA	3.73 ± 0.41 aA	2.91 ± 0.38 bcA	3.65 ± 0.34 abA
Palisade parenchyma thickness (μm)	137.98 ± 6.22 dA	170.96 ± 4.62 cA	229.48 ± 9.56 aA	193.13 ± 8.77 bA
Spongy parenchyma thickness (μm)	67.85 ± 6.37 bA	72.73 ± 4.02 abA	81.54 ± 7.04 aA	82.67 ± 7.36 aA
Palisade tissue/Spongy tissue	2.09 ± 0.20 bA	2.38 ± 0.16 bA	2.89 ± 0.27 aA	2.37 ± 0.14 bA
Midvein xylem cross-sectional area (μm^2^)	55,072.68 ± 3904.62 cA	64,644.26 ± 2642.99 bA	79,649.74 ± 6764.80 aA	27,635.20 ± 3390.20 dA
Midvein vascular bundle area (μm^2^)	83,002.15 ± 2811.54 cA	100,758.92 ± 2858.79 bA	119,199.70 ± 6550.88 aA	41,334.44 ± 5482.73 dA
Midvein phloem cross-sectional area (μm^2^)	27,929.48 ± 3159.99 bA	36,114.66 ± 1384.62 aA	39,549.97 ± 3031.85 aA	13,699.25 ± 2431.73 cB
Ratio of palisade/leaf thickness	0.44 ± 0.03 bAB	0.44 ± 0.03 bB	0.55 ± 0.06 aA	0.51 ± 0.06 aA
Ratio of spongy tissue/leaf thickness	0.22 ± 0.04 aC	0.19 ± 0.02 aC	0.20 ± 0.04 aB	0.22 ± 0.03 aB

All experiments were performed with eight replicates. The data in the table are mean values ± standard deviation; different lowercase letters indicate significant differences (*p* < 0.05) among different stress treatments for the same tree species at the same stress time; different uppercase letters indicate significant differences (*p* < 0.05) among the same stress treatment for different tree species at the same stress time. CK represents the well-watered treatment (control), T1 represents mild drought stress, T2 represents moderate drought stress, and T3 represents severe drought stress.

**Table 2:** Effects of drought stress on seedling microanatomical structure of *E. angustifolia*.

Index	*E. angustifolia*			
	CK	T1	T2	T3
Leaf lamina thickness (μm)	175.66 ± 15.87 bB	211.46 ± 12.86 aB	187.44 ± 16.07 abB	210.49 ± 18.51 aB
Midvein thickness (μm)	340.60 ± 32.58 bC	420.63± 15.11 aB	326.78 ± 26.18 bB	396.43 ± 15.99 aB
Upper epidermal thickness (μm)	13.32 ± 1.09 bB	15.56 ± 0.74 aB	13.53 ± 1.01 abB	13.08 ± 1.21 bB
Lower epidermal thickness (μm)	7.07 ± 0.57 abC	7.16 ± 0.69 abC	6.56 ± 0.44 bC	7.82 ± 0.44 aC
Upper stratum corneum thickness (μm)	3.61 ± 0.45 bA	4.03 ± 0.24 abA	4.49 ± 0.49 aA	3.38 ± 0.22 bA
Lower stratum corneum thickness (μm)	2.23 ± 0.17 aAB	2.41 ± 0.19 aB	2.22 ± 0.22 aB	2.04 ± 0.25 aB
Palisade parenchyma thickness (μm)	73.93 ± 5.03 bB	99.28 ± 9.90 aB	88.16 ± 9.81 abB	94.72 ± 10.04 aB
Spongy parenchyma thickness (μm)	43.20 ± 3.60 bB	50.54 ± 2.68 aB	45.08 ± 2.44 abB	49.59 ± 3.52 abB
Palisade tissue/Spongy tissue	1.73 ± 0.08 aB	1.96 ± 0.13 aB	1.95 ± 0.18 aB	1.90 ± 0.15 aB
Midvein xylem cross-sectional area (μm^2^)	21,190.27 ± 3573.08 aB	17,762.69 ± 2033.14 abC	13,518.42 ± 3537.65 bC	17,860.10 ± 2443.53 abB
Midvein vascular bundle area (μm^2^)	41,343.92 ± 5140.08 aB	43,302.22 ± 6057.82 aB	28,522.47 ± 6449.16 bC	37,057.22 ± 4094.79 abA
Midvein phloem cross-sectional area (μm^2^)	20,153.66 ± 1662.58 abB	25,539.53 ± 4241.89 aB	15,004.05 ± 3142.88 bA	19,197.13 ± 1919.03 bA
Ratio of palisade/leaf thickness	0.43 ± 0.03 aB	0.47 ± 0.05 aB	0.47 ± 0.04 aB	0.45 ± 0.03 aB
Ratio of spongy tissue/leaf thickness	0.25 ± 0.03 aB	0.24 ± 0.02 aB	0.24 ± 0.03 aA	0.24 ± 0.04 aB

All experiments were performed with eight replicates. The data in the table are mean values ± standard deviation. Different lowercase letters indicate significant differences (*p* < 0.05) among different stress treatments for the same tree species at the same stress time; different uppercase letters indicate significant differences (*p* < 0.05) among the same stress treatment for different tree species at the same stress time. CK represents the well-watered treatment (control), T1 represents mild drought stress, T2 represents moderate drought stress, and T3 represents severe drought stress.

**Table 3:** Effects of drought stress on seedling microanatomical structure of *X. sorbifolium*.

Index	*X. sorbifolium*			
	CK	T1	T2	T3
Leaf lamina thickness (μm)	175.43 ± 2.52 aB	161.76 ± 5.31 aC	169.68 ± 5.16 aB	140.16 ± 16.90 bC
Midvein thickness (μm)	404.36 ± 31.22 aB	347.37 ± 8.23 abC	359.97 ± 5.02 bB	318.36 ± 19.93 cC
Upper epidermal thickness (μm)	16.49 ± 1.81 aB	14.84 ± 0.76 abB	13.38 ± 0.68 bB	13.80 ± 1.17 bB
Lower epidermal thickness (μm)	11.61 ± 0.82 aB	10.57 ± 1.75 abB	10.64 ± 0.48 abB	9.84 ± 0.61 bB
Upper stratum corneum thickness (μm)	2.19 ± 0.22 aB	2.01 ± 0.17 abB	2.06 ± 0.14 abB	1.84 ± 0.06 bB
Lower stratum corneum thickness (μm)	1.80 ± 0.14 aB	1.46 ± 1.69 bC	1.84 ± 0.15 aB	1.33 ± 0.18 bC
Palisade parenchyma thickness (μm)	84.68 ± 6.46 aB	85.08 ± 3.67 aC	95.63 ± 2.20 aB	59.34 ± 10.61 bC
Spongy parenchyma thickness (μm)	48.61 ± 0.61 aB	48.01 ± 1.07 aB	43.59 ± 2.11 aB	47.60 ± 4.25 aB
Palisade tissue/Spongy tissue	48,760.02 ± 7304.13 aA	29,189.96 ± 2539.05 bB	31,184.39 ± 1676.17 bB	19,847.32 ± 3584.63 cB
Midvein xylem cross-sectional area (μm^2^)	70,154.88 ± 10678.04 aA	42,988.90 ± 3695.66 bcB	50,928.59 ± 1047.99 bB	31,658.24 ± 5564.35 cA
Midvein vascular bundle area (μm^2^)	21,394.86 ± 4401.84 aAB	13,798.94 ± 1774.63 bC	19,744.20 ± 1450.00 aB	11,810.92 ± 2221.53 bB
Midvein phloem cross-sectional area (μm^2^)	0.48 ± 0.07 Ab	0.53 ± 0.02 abA	0.56 ± 0.04 aA	0.41 ± 0.06 cB
Ratio of palisade/leaf thickness	0.28 ± 0.01 bcA	0.30 ± 0.03 bA	0.26 ± 0.02 cA	0.35 ± 0.05 aA

All experiments were performed with eight replicates. The data in the table are mean values ± standard deviation. Different lowercase letters indicate significant differences (*p* < 0.05) among different stress treatments for the same tree species at the same stress time; different uppercase letters indicate significant differences (*p* < 0.05) among the same stress treatment for different tree species at the same stress time. CK represents the well-watered treatment (control), T1 represents mild drought stress, T2 represents moderate drought stress, and T3 represents severe drought stress.

## Data Availability

The data presented in this study are available on request from the corresponding author due to privacy/ethical restrictions.
